# Short-Term Outcomes After Total Shoulder Arthroplasty in Octogenarians: A Matched Analysis

**DOI:** 10.7759/cureus.16441

**Published:** 2021-07-17

**Authors:** John Carney, Erik Gerlach, Mark A Plantz, Colin Cantrell, Peter R Swiatek, Jeremy S Marx, Guido Marra

**Affiliations:** 1 Department of Orthopaedic Surgery, Northwestern University Feinberg School of Medicine, Chicago, USA; 2 Department of Orthopaedic Surgery, Northwestern Memorial Hospital, Chicago, USA

**Keywords:** octogenarian, total shoulder arthroplasty, outcomes, risk factors, osteoarthritis, complications

## Abstract

Introduction

Studies have shown that the use of total shoulder arthroplasty is increasing every year in the United Stated at a rate higher than that of total hip or total knee arthroplasty. As the population of the United States continues to age, it is becoming more important for surgeons to understand the true impact of age on outcomes and complications following procedures such a total shoulder arthroplasty. The purpose of this study was to determine if octogenarians have poorer outcomes after total shoulder arthroplasty compared to a younger, matched control group.

Methods

Data was obtained through the American College of Surgeons National Surgical Quality Improvement Program database (ACS NSQIP). Patients who had undergone total shoulder arthroplasty were identified by Current Procedural Terminology code (23472). Indication for arthroplasty was determined by ICD9/10 code (osteoarthritis, fracture, other). Each octogenarian was matched 1:1 to a non-octogenarian based on sex, BMI, ASA class, medical comorbidities, functional status, and surgical indication for arthroplasty by propensity scoring. A subgroup analysis was performed to compare outcomes between only those patients who underwent TSA for osteoarthritis.Outcomes of interest were assessed between the two groups for statistical significance using a chi-squared test or fisher exact test for expected values of less than 5. Statistical significance was set at p<0.05.

Results

After matching, octogenarians were found to be at higher risk of readmission (4.7% vs. 3.3%, p=0.046), non-home discharge (27.1% vs. 9.4%, p<0.001), and overall surgical (4.4% vs. 2.5%, p=0.006) and medical complications (3.7% vs. 2.4%, p=0.039). In the setting of TSA for osteoarthritis only, however, octogenarians were only at higher risk for non-home discharge (22.4% vs. 7.5%, p<0.001).

Conclusions

Octogenarians are at higher risk of some complications following total shoulder arthroplasty but fewer than has been previously reported, particularly in the setting of arthroplasty for the treatment of arthritis.

## Introduction

Total shoulder arthroplasty (TSA) has been well established as an efficacious and valid treatment modality for a myriad of glenohumeral joint pathologies [1-4]. The incidence of TSA has been growing annually in the United States and has currently outpaced that of total hip or total knee arthroplasty [5]. TSA is performed using one of two techniques. The first is the anatomic TSA technique which employs implants resembling native shoulder anatomy. The second being the reverse total shoulder arthroplasty (RTSA) technique that can function in the setting of rotator cuff insufficiency. This approach utilizes implants that places the ball component on the glenoid and the socket component to the proximal humerus [6]. A vast body of evidence shows that both TSA techniques have better radiographic and patient reported outcomes in comparison to other surgical treatment modalities for conditions such as proximal humerus fractures and glenohumeral arthritis [2,3,7-9].

While TSA can provide excellent outcomes for patients with a variety of glenohumeral pathologies, the procedure is not risk free. Periprosthetic fracture, implant failure, peri-operative medical complications and infection are all serious complications of TSA that can lead to reoperation, diminished functional results, or even death if not managed in a timely and appropriate manner [10,11]. Hence, it is crucial that surgeons be meticulous in selecting appropriate patients for TSA and remain aware of risk factors for poor outcomes and complications.

Age has often been identified as a risk factor for complication throughout the orthopaedic literature [11-14]. However, this risk factor is non-modifiable, and with an aging population in the United States, there is a need for surgeons to better understand the impact of age on orthopaedic outcomes. This is particularly true in the setting of arthroplasty, which typically is performed in older populations. Octogenarians (i.e. individuals of age 80 or older) have been the subject of numerous outcome studies in arthroplasty which have shown this group to be at a higher risk of complication [11-13,15,16]. The purpose of this study was to determine if octogenarians have poorer outcomes after total shoulder arthroplasty compared to a younger, matched control group.

## Materials and methods

Data was obtained through the American College of Surgeons National Surgical Quality Improvement Program database (ACS NSQIP). This database is collected by a group of trained surgical clinical reviewers, who record perioperative data at over 700 hospitals across the United States, including International Classification of Disease 9th and/or 10th Revisions (ICD-9, ICD-10) codes, Current Procedural Terminology (CPT) codes, and data related to discharge disposition, reoperations, readmissions, and mortality through 30 days postoperatively [17]. This database is considered among the most precise and accurate database available for measuring patient outcomes after surgery given its high rate of complete data sets, operative data points, validation, and inter-rater reliability [18].

Patients who had undergone total shoulder arthroplasty were identified by CPT code (23472). Indication for arthroplasty was determined by ICD9/10 code (osteoarthritis, fracture, other). Patients were divided into two groups: octogenarians (80+ years old) and control (age 65-79). Both elective and non-elective procedures were included.

For the control group, each octogenarian was matched 1:1 to a non-octogenarian based on sex, BMI, ASA class, medical comorbidities, functional status, and surgical indication for arthroplasty. Matching was performed using propensity scoring. A subgroup analysis was performed to compare outcomes between only those patients who underwent TSA for a primary diagnosis of osteoarthritis.

Outcomes of interest included return to the operating room, readmission, reoperation, non-home discharge, mortality, surgical complications (superficial surgical site infection, deep surgical site infection, dehiscence, bleeding), medical complications (wound infection, pneumonia, reintubation, failure to wean intubation, pulmonary embolism, renal insufficiency, renal failure, urinary tract infection, cerebrovascular accident, cardiac arrest, myocardial infarction, deep venous thromboembolism, systemic sepsis, and septic shock). Differences in these outcomes were assessed between the two groups for statistical significance using a chi-squared test or fisher exact test for expected values of less than 5. Statistical significance was set at p<0.05. All statistical calculations were performed using IBM SPSS Version 24 (Armonk, NY; IBM Corp).

## Results

A total of 8,382 patients were eligible for inclusion in the study. Of these, 1,625 were octogenarians (mean age +/- SD) while the other 6,757 were aged 65-79 (mean age +/- SD). Prior to matching, the octogenarian group had lower rates of obesity (35.1% vs. 51.2%, p<0.001), diabetes (15.9% vs. 20.1%, p<0.001), and smoking (2.8% vs. 7.2%, p<0.001). The octogenarian group had higher rates of COPD (8.7% vs 6.9%, p=0.012), CHF (1.2% vs 0.6%, p=0.004), hypertension (78.2% vs 71.1%, p<0.001), bleeding disorders (4.4% vs 2.4%, p<0.001), and transfusions within 48 hours of surgery (0.7% vs 0.2%, p=0.001). They were also less likely to be functionally independent (94.6% vs 97.5%, p<0.001) and belong to an ASA class of 3 or greater (64.6% vs 56.1%, p<0.001). Lastly, octogenarians were more likely to undergo shoulder arthroplasty for fracture (11.6% vs. 5.8%, p<0.001) than osteoarthritis (48.3% vs 55.0%, p<0.001) compared to the control group (p<0.05). Findings are summarized in Table 1.

**Table 1:** Demographics, patient variables, comorbidities, and surgical indication prior to matching

	65-79 years old		80+ years old		p
	[n = 6,757]		[n = 1,625]		
Sex					
Male	2,827	41.80%	512	31.50%	<0.001
Female	3,930	58.20%	1,113	68.50%	
BMI (kg/m^2^)					
Underweight	82	1.20%	29	1.80%	0.071
Normal	1,012	15.00%	396	24.40%	<0.001
Overweight	2,205	32.60%	630	38.80%	<0.001
Obese Class I	1,796	26.60%	393	24.20%	0.048
Obese Class II	944	14.00%	122	7.50%	<0.001
Obese Class III	718	10.60%	55	3.40%	<0.001
Comorbidities					
Diabetes	1,359	20.10%	259	15.90%	<0.001
Smoking	486	7.20%	45	2.80%	<0.001
COPD	468	6.90%	142	8.70%	0.012
Congestive Heart Failure	38	0.60%	20	1.20%	0.004
Hypertension	4,801	71.10%	1,270	78.20%	<0.001
Renal Failure	5	0.10%	0	0.00%	0.591
Chronic Steroid Use	342	5.10%	75	4.60%	0.458
Bleeding Disorder	162	2.40%	72	4.40%	<0.001
Transfusion within 48 hours	12	0.20%	11	0.70%	0.001
Functional Status					
Independent	6,591	97.50%	1,538	94.60%	<0.001
Non-independent	166	2.50%	87	5.40%	
Surgical Indication					
Fracture	389	5.80%	189	11.60%	<0.001
Osteoarthritis	3,717	55.00%	785	48.30%	<0.001
Other	2,651	39.20%	651	40.10%	0.54
ASA Class					
Class 1 (No disturbance)	71	1.10%	3	0.20%	<0.001
Class 2 (Mild disturbance)	2,704	40.00%	499	30.70%	<0.001
Class 3 (Severe disturbance)	3,788	56.10%	1,050	64.60%	<0.001
Class 4+ (Life threatening)	186	2.80%	70	4.30%	0.001

Prior to matching, the octogenarian group had higher rates of readmission (5.2% vs 2.8%, p<0.001), non-home discharge (30.0% vs 9.9%, p<0.001), mortality (0.5% vs 0.1%, p<0.001), surgical complications (5.4% vs 2.4%, p<0.001), and medical complications (3.9% vs 2.1%, p<0.001). Regarding surgical complications specifically, the octogenarians had higher rates of bleeding (5.4% vs. 2.0%, p<0.001). Regarding medical complications, specifically, the octogenarians had higher rates of pneumonia (1.0% vs 0.5%, p=0.024), reintubation (0.5% vs. 0.2%, p=0.029), failure to wean intubation (0.4% vs 0.1%, p=0.007), pulmonary embolism (0.6% vs 0.3%, p=0.020), urinary tract infection (UTI) (1.5% vs 0.6%, p<0.001), cardiac arrest (0.2% vs 0.0%, p=0.015), and DVT (0.6% vs 0.3%, p=0.029). Findings are summarized in Table 2.

**Table 2:** 30-day outcome measures and complication rates prior to matching

	65-79 years old		80+ years old		p
	[n = 6,757]		[n = 1,625]		
Return to OR	82	1.20%	26	1.60%	0.215
Readmission	191	2.80%	85	5.20%	<0.001
Reoperation	82	1.20%	26	1.60%	0.215
Non-home discharge	671	9.90%	488	30.00%	<0.001
Mortality	6	0.10%	8	0.50%	<0.001
Surgical Complications					
Overall	161	2.40%	88	5.40%	<0.001
Superficial surgical site infection	10	0.10%	1	0.10%	0.703
Deep surgical site infection	12	0.20%	1	0.10%	0.484
Dehiscence	1	0.00%	0	0.00%	>0.999
Bleeding	138	2.00%	87	5.40%	<0.001
Medical Complications					
Overall	144	2.10%	64	3.90%	<0.001
Wound infection	3	0.00%	0	0.00%	>0.999
Pneumonia	34	0.50%	16	1.00%	0.024
Reintubation	13	0.20%	8	0.50%	0.029
Failure to wean intubation	6	0.10%	6	0.40%	0.007
Pulmonary embolism	17	0.30%	10	0.60%	0.02
Renal insufficiency	5	0.10%	2	0.10%	0.627
Renal failure	2	0.00%	2	0.10%	0.171
Urinary tract infection	41	0.60%	25	1.50%	<0.001
Cerebrovascular accident	6	0.10%	3	0.20%	0.389
Cardiac arrest	2	0.00%	4	0.20%	0.015
Myocardial infarction	23	0.30%	6	0.40%	0.859
Deep venous thromboembolism	18	0.30%	10	0.60%	0.029
Systemic sepsis	10	0.10%	5	0.30%	0.171
Septic shock	2	0.00%	3	0.20%	0.053

Propensity scoring resulted in the groups being well matched (p>0.99 for all demographic and preoperative metrics) (Table 3). Compared to the matched control group, octogenarians continued to demonstrate significantly higher rates of readmission (4.7% vs. 3.3%, p=0.046) and non-home discharge (27.1% vs 9.4%, p<0.001). They also continued to demonstrate higher rates of overall surgical complications (4.4% vs 2.5%, p=0.006) and bleeding (4.3% vs 2.2%, p=0.001) as well as overall medical complications (3.7% vs 2.4%, p=0.039). However, they no longer demonstrated statistically different rates of mortality or any specific medical complication (Table 4). Subgroup analysis of octogenarians undergoing shoulder arthroplasty for a diagnosis of osteoarthritis only demonstrated a significantly higher rate of non-home discharge (22.4% vs 7.5%, p<0.001) (Table 5).

**Table 3:** Demographics, patient variables, comorbidities, and surgical indication after matching

	65-79 years old		80+ years old		p
	[n = 1,434]		[n = 1,434]		
Sex					
Male	454	31.70%	454	31.70%	>0.999
Female	980	68.30%	980	68.30%	
BMI (kg/m^2^)					
Underweight	16	1.10%	16	1.10%	>0.999
Normal	328	22.90%	328	22.90%	>0.999
Overweight	575	40.10%	575	40.10%	>0.999
Obese Class I	355	24.80%	355	24.80%	>0.999
Obese Class II	112	7.80%	112	7.80%	>0.999
Obese Class III	48	3.30%	48	3.30%	>0.999
Comorbidities					
Diabetes	222	15.50%	222	15.50%	>0.999
Smoker	33	2.30%	33	2.30%	>0.999
COPD	91	6.30%	91	6.30%	>0.999
Congestive Heart Failure	1	0.10%	1	0.10%	>0.999
Hypertension	1,127	78.60%	1,127	78.60%	>0.999
Renal Failure	0	0.00%	0	0.00%	>0.999
Chronic Steroid Use	47	3.30%	47	3.30%	>0.999
Bleeding Disorder	35	2.40%	35	2.40%	>0.999
Transfusion within 48 hours	1	0.10%	1	0.10%	>0.999
Functional status					
Independent	1,404	97.90%	1,404	97.90%	>0.999
Non-independent	30	2.10%	30	2.10%	>0.999
Surgical Indication					
Fracture	119	8.30%	119	8.30%	>0.999
Osteoarthritis	731	51.00%	731	51.00%	>0.999
Other	584	40.70%	584	40.70%	>0.999
ASA Class					
Class 1 (No disturbance)	3	0.20%	3	0.20%	>0.999
Class 2 (Mild disturbance)	471	32.80%	471	32.80%	>0.999
Class 3 (Severe disturbance)	937	65.30%	937	65.30%	>0.999
Class 4+ (Life threatening)	23	1.60%	23	1.60%	>0.999

**Table 4:** 30-day outcome measures and complication rates after matching

	65-79 years old		80+ years old		p
	[n = 1,434]		[n = 1,434]		
Return to OR	19	1.30%	22	1.50%	0.637
Readmission	47	3.30%	68	4.70%	0.046
Reoperation	19	1.30%	22	1.50%	0.637
Non-home discharge	135	9.40%	389	27.10%	<0.001
Mortality	1	0.10%	6	0.40%	0.125
Surgical Complications					
Overall	36	2.50%	63	4.40%	0.006
Superficial surgical site infection	2	0.10%	1	0.10%	>0.999
Deep surgical site infection	3	0.20%	1	0.10%	0.625
Dehiscence	0	0.00%	0	0.00%	–
Bleeding	31	2.20%	62	4.30%	0.001
Medical Complications					
Overall	34	2.40%	53	3.70%	0.039
Wound infection	0	0.00%	0	0.00%	–
Pneumonia	8	0.60%	13	0.90%	0.273
Reintubation	4	0.30%	7	0.50%	0.548
Failure to wean intubation	2	0.10%	6	0.40%	0.288
Pulmonary embolism	3	0.20%	9	0.60%	0.145
Renal insufficiency	1	0.10%	2	0.10%	>0.999
Renal failure	0	0.00%	2	0.10%	0.5
Urinary tract infection	10	0.70%	20	1.40%	0.066
Cerebrovascular accident	1	0.10%	3	0.20%	0.625
Cardiac arrest	0	0.00%	3	0.20%	0.25
Myocardial infarction	6	0.40%	5	0.30%	0.763
Deep venous thromboembolism	2	0.10%	8	0.60%	0.109
Systemic sepsis	4	0.30%	4	0.30%	>0.999
Septic shock	1	0.10%	3	0.20%	0.625

**Table 5:** 30-day outcome measures and complication rates after elective rTSA for osteoarthritis: matched comparison

	65-79 years old		80+ years old		p
	[n = 731]		[n = 731]		
Return to OR	8	1.10%	9	1.20%	0.807
Readmission	17	2.30%	22	3.00%	0.417
Reoperation	8	1.10%	9	1.20%	0.807
Non-home discharge	55	7.50%	164	22.40%	<0.001
Mortality	0	0.00%	4	0.50%	0.124
Surgical Complications					
Overall	11	1.50%	15	2.10%	0.429
Superficial surgical site infection	1	0.10%	0	0.00%	>0.999
Deep surgical site infection	1	0.10%	0	0.00%	>0.999
Dehiscence	0	0.00%	0	0.00%	–
Bleeding	9	1.20%	15	2.10%	0.217
Medical Complications					
Overall	15	2.10%	21	2.90%	0.311
Wound infection	0	0.00%	0	0.00%	–
Pneumonia	4	0.50%	4	0.50%	>0.999
Reintubation	1	0.10%	2	0.30%	>0.999
Failure to wean intubation	1	0.10%	3	0.40%	0.624
Pulmonary embolism	1	0.10%	5	0.70%	0.218
Renal insufficiency	1	0.10%	2	0.30%	>0.999
Renal failure	0	0.00%	0	0.00%	–
Urinary tract infection	5	0.70%	6	0.80%	0.762
Cerebrovascular accident	1	0.10%	1	0.10%	>0.999
Cardiac arrest	0	0.00%	1	0.10%	>0.999
Myocardial infarction	2	0.30%	2	0.30%	>0.999
Deep venous thromboembolism	0	0.00%	2	0.30%	0.5
Systemic sepsis	3	0.40%	0	0.00%	0.249
Septic shock	1	0.10%	1	0.10%	>0.999

## Discussion

The purpose of this study was to determine if octogenarians have inferior short-term outcomes after total shoulder arthroplasty compared to a younger, matched control group. After matching, our data shows that octogenarians are at higher risk of readmission, non-home discharge, and surgical and medical complication. In the setting of TSA for osteoarthritis, however, octogenarians do not have a higher rate of readmission or complication but continue to have higher rates of non-home discharge.

To our knowledge there are two other studies that has investigated TSA in octogenarians. One study similarly found a higher overall complication rate in octogenarians. However, this group also found that octogenarians were at a higher risk of several, specific major and minor complications within 30 days including pulmonary embolism, stroke, myocardial infarction, reintubation and septic shock [11]. The differences between our results and the aforementioned study are likely due to the fact that the aforementioned study did not create two equal sized groups, but rather compared all TSA patients in the database for a set time period. Like in our sample, the octogenarians and non-octogenarians in this study had several significant differences in demographics and comorbidities. The aforementioned study accounted for these differences by utilizing a logistic regression model whereas our study created a 1:1 matched control group via propensity scoring, which may better account for differences in covariates among two groups and explain why we did not find octogenarians to be at a higher risk for these major complications. The other study also utilized the same database as ours and found that octogenarians were at a higher risk for readmission, pneumonia, and UTI compared to individuals younger than 70 years old. They were not, however, at a higher risk for any complications than individuals aged 70-80 [19].

Several studies have been conducted on octogenarians in the hip, knee, and ankle arthroplasty fields that are in agreement with our findings. A recent database study of octogenarians undergoing ankle arthroplasty found the group to be at a higher risk for non-home discharge and longer hospital length of stay but not at an increased risk for higher complication [12]. A 2016 review article that compiled morbidity and mortality data from 11 different studies on hip and knee arthroplasty in octogenarians and nonagenarians concluded that although advanced age patients have more medical comorbidities, these comorbidities do not translate to a higher rate of surgical complication, though some studies did note higher rates of medical complications [20-26]. Although our study did find a higher rate of complications in octogenarians, this finding became statistically insignificant when TSA patients without a primary diagnosis of osteoarthritis were removed from the dataset, which is likely a more similar patient population to the hip and knee arthroplasty populations. Some studies have found longer lengths of stay in this population following arthroplasty. More recent literature in advanced age patients, however, has not found this to be the case, which can likely be attributed to improved protocols involving co-management of older patients with geriatricians [20,24].

Similar findings have also been found when assessing the revision arthroplasty in octogenarians. A 2007 study investigating outcomes in revision hip arthroplasty patients over the age of 80 compared to a control group found equivalent patient reported outcome scores and no difference in complications other than dislocation, which was higher in the younger control group [27]. A 2018 study investigating total knee arthroplasty in octogenarians compared to a propensity score matched control group found higher rate of blood transfusion and length of stay, but no increases in any other medical or surgical complication nor mortality or readmission [13].

This study has several weaknesses which should be considered when interpreting our findings. First, this study utilizes a database which can be subject to input errors and can only provide a limited number of metrics regarding a patient’s medical care. The database does not provide specific information regarding severity of comorbidities or specific outcomes related to a given procedure. Access to the database is limited to contributing institutions, and the high cost of participation has led to a disproportionate contribution to the NSQIP from large teaching hospitals [18,28]. Second, the database identifies all-cause readmission, not just orthopedic-related readmissions. Third, there are no separate CPT codes for anatomic total shoulder arthroplasty or reverse total shoulder arthroplasty, and thus we are not able to identify whether the groups were equally matched regarding the type of arthroplasty that was performed, or if one form of arthroplasty demonstrated lower complication rates.

This study contributes to the current body of literature as it presents valuable information to surgeons on the increased risk that age alone presents when performing total shoulder arthroplasty, and can help surgeons make appropriate decisions regarding how to care for patients of this age group. Further, a prospective study is warranted to better quantify what factors are predictive of better or poorer outcomes in this population.

## Conclusions

Octogenarians are at higher risk of readmission, non-home discharge, and surgical and medical complications following total shoulder arthroplasty. However, this age group is only at risk of non-home discharge when TSA is being performed for the diagnosis of arthritis. This information will assist surgeons with appropriately selecting surgical candidates in their practice and anticipating potential challenges and complications in the early post-operative period.
